# Tissue-Level Regeneration and Remodeling Dynamics are Driven by Mechanical Stimuli in the Microenvironment in a Post-Bridging Loaded Femur Defect Healing Model in Mice

**DOI:** 10.3389/fcell.2022.856204

**Published:** 2022-05-24

**Authors:** Graeme R. Paul, Paul Vallaster, Michelle Rüegg, Ariane C. Scheuren, Duncan C. Tourolle, Gisela A. Kuhn, Esther Wehrle, Ralph Müller

**Affiliations:** Institute for Biomechanics, ETH Zurich, Zurich, Switzerland

**Keywords:** bone, mechanobiology, fracture healing, adaptive loading, real-time finite element analysis, microenvironment, multi-density analysis

## Abstract

Bone healing and remodeling are mechanically driven processes. While the generalized response to mechanical stimulation in bone is well-understood, much less is known about the mechanobiology-regulating tissue-scale bone formation and resorption during the reparative and remodeling phases of fracture healing. In this study, we combined computational approaches in the form of finite element analysis and experimental approaches by using a loaded femoral defect model in mice to investigate the role of mechanical stimulation in the microenvironment of bone. Specifically, we used longitudinal micro-computed tomography to observe temporal changes in bone at different densities and micro-finite element analysis to map the mechanics of the microenvironment to tissue-scale formation, quiescence (no change in bone presence between time points), and resorption dynamics in the late reparative and remodeling phases (post bridging). Increasing levels of effective strain led to increasing conditional probability of bone formation, while decreasing levels of effective strain led to increasing probability of bone resorption. In addition, the analysis of mineralization dynamics showed both a temporal and effective strain level-dependent behavior. A logarithmic-like response was displayed, where the conditional probability of bone formation or resorption increased rapidly and plateaued or fell rapidly and plateaued as mechanical strain increased.

## Introduction

The mechanobiological association between bone healing (Augat et al., 1998; Carter et al., 1998; Bailón-Plaza and van der Meulen, 2003; Augat et al., 2005; Morgan et al., 2008; Morgan et al., 2010; Tourolle né Betts et al., 2020), bone remodeling (Webster et al., 2012; Schulte et al., 2013; Birkhold et al., 2014; Meakin et al., 2014; Scheuren et al., 2020), and mechanical stimulation is well-established. Many authors have shown mechano-regulatory behavior at the organ and tissue scale in bone remodeling models (Webster et al., 2008; Schulte et al., 2013; Lambers et al., 2015; Webster et al., 2015; Scheuren et al., 2020). However, in bone healing, the influence of mechanical stimulation throughout all the three phases of fracture healing lacks a thorough understanding at the tissue scale (Tourolle né Betts et al., 2020). Improved knowledge of the effects of mechanics in the microenvironment on all phases of fracture healing will allow better understanding of fixation methods, biomaterial application, and pharmacological effects on mechanosensitive cells.

Fracture healing displays three overlapping phases, which are as follows: inflammation, repair, and remodeling (Marsell and Einhorn, 2011). Almost immediately after fracture, which is called the inflammatory phase, a hematoma forms. Following this, the early reparative phase begins, and lowly mineralized tissue starts forming. The term lowly in this context refers to a low degree of mineralization of the bone tissue (i.e., the opposite of highly mineralized bone). The fracture bridges in the reparative phase, and the lowly mineralized tissue begins to mineralize, overshooting the required amount of bone needed for structural stability. This excess bone is removed in the remodeling stage (Ghiasi et al., 2017). Similar to the studies on bone remodeling, micro-computed tomography (micro-CT) has allowed the longitudinal quantification of this process (Morgan et al., 2009; Wehrle et al., 2019a). More recently, micro-finite element analysis (micro-FE) has also been used to link mechanical stimuli to the patterns of formation, resorption, and quiescence during the fracture healing process (Morgan et al., 2010; Tourolle né Betts et al., 2020), indicating that soft- and bone-tissue strains allow improved prediction of where bone will form. This echoes what is seen in bone remodeling studies, where several authors have coupled micro-CT, micro-FE, and cyclic mechanical loading to show that the tissue-scale changes are correlated with the mechanical microenvironment (Lambers et al., 2013; Schulte et al., 2013; Lambers et al., 2015; Scheuren et al., 2020). More specifically, high local strains within the mature and mineralized bone tissue have been shown to increase the likelihood of site-specific bone formation, whereas sites of resorption correlated with low local strains (Carter et al., 1998; Claes et al., 1998; Claes and Heigele, 1999; Schulte et al., 2013; Lambers et al., 2015; Scheuren et al., 2020). Building on investigations in mature bone, Tourolle né Betts et al. (2020) developed a multi-density approach, whereby a range of densities was analyzed to investigate the link between mechanics and mineralization dynamics in lowly mineralized woven bone. While the initial periods of the inflammation and reparative phases show limited similarities between fracture healing and bone remodeling, the late reparative and remodeling phases that occur after bridging should have much in common (Huiskes et al., 2000).

The combined experimental and computational approach of micro-CT and micro-FE are well-established tools for investigating bone adaptation (Webster et al., 2008; Bouxsein et al., 2010; Schulte et al., 2011; Lambers et al., 2013; Schulte et al., 2013; Lambers et al., 2015; Webster et al., 2015; Paul et al., 2018), and many different approaches have been taken to describe the mechanical environment. Currently, the main mechanism driving cell response to mechanical stimuli is debated, with direct cellular strain and indirect fluid shear stresses being supported by several studies (Fritton and Weinbaum, 2009; Weinbaum et al., 2011; Klein-Nulend et al., 2013). To combine these mechanisms, SED is often used (Webster et al., 2012; Schulte et al., 2013; Webster et al., 2015; Scheuren et al., 2020) as it combines volumetric and deviatoric strains (which drive fluid movement and direct strain, respectively). However, SED scales linearly with material stiffness, and hence, while it is an appropriate metric for mature bone, it has limitations for rapidly mineralizing tissue found in bone healing (Tourolle né Betts et al., 2020). Hence, effective strain has been used by several authors (Pistoia et al., 2002; Tourolle né Betts et al., 2020). Since effective strain combines volumetric and deviatoric strains, it allows for better comparison of bone remodeling and healing (Tourolle né Betts et al., 2020) than SED.

*In silico* models can aid understanding of the mechanobiology of bone remodeling and healing (Ghiasi et al., 2019). In particular, they allow rapid parameter investigation (Lacroix and Prendergast, 2002), forming the foundation for more targeted experiments. Often, these models use simplified or mathematically derived relationships (Isaksson et al., 2007) to describe the mechano-regulation of bone healing. There exists a lack of accurate, experimentally derived data to quantify the exact relationship between the microenvironment and tissue-scale changes in the late reparative and remodeling phases of fracture healing (Geris et al., 2009). This limits the accuracy of the mechano-regulatory aspect of *in silico* modeling in fracture healing. Quantification of this relationship will allow improved mechano-regulatory descriptions in *in silico* models of bone healing.

In this study, we analyze the sites of formation, resorption, and quiescence determined *via* an experimental approach of longitudinal *in vivo* micro-CT and couple them with a computational approach of micro-FE analysis to investigate the role played by effective strain within the microenvironment in the late reparative and remodeling phases of a loaded femur defect healing model. We incorporate a multidensity approach to allow analysis of bone tissue formation and mineralization under mechanical stimulation and perform a correlative analysis into the mechanoresponsivity of bone during fracture healing. We hypothesize that late phases of fracture healing display similar mechano-regulatory behavior to bone remodeling. More specifically, we hypothesize that mechanoresponsivity will be greater in the mechanically loaded group and that both the physiological (sham-loaded/control) group and the extra-physiologically loaded group will have greater probability of site-specific formation and resorption in regions of higher and lower effective strain, respectively. Determining these relationships will provide a foundation for realistic rules for *in silico* investigations of bone during the post-bridging phases of fracture healing and improve our understanding of mechanobiological relationships in fracture healing.

## Results

**FIGURE 1:**
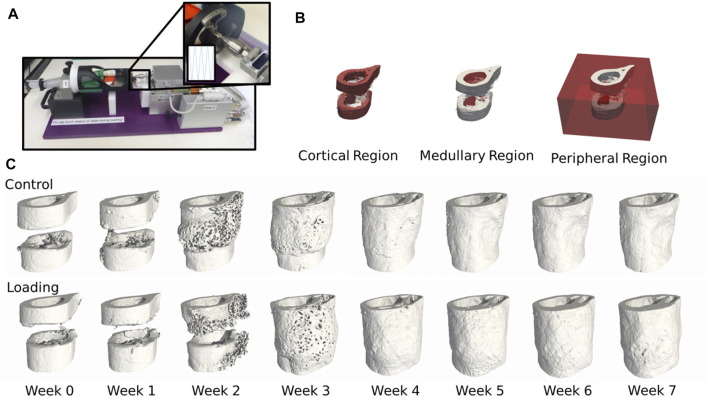
**(A)** Femur defect loading was performed by an electromagnetic actuator, a specially designed holder, and an external fixator (Wehrle et al., 2021). **(B)** Three mask regions at week 0 of the femur defect regions. **(C)** Temporal progression of femur defect healing of both control and loaded mice.

**FIGURE 2:**
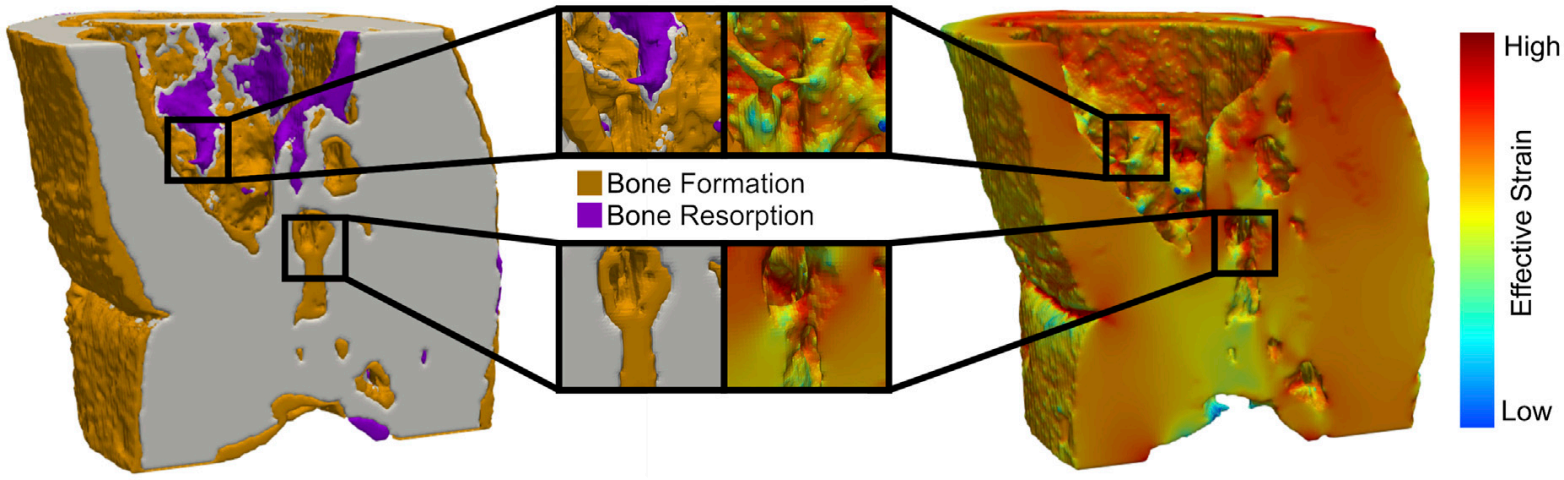
Regions of high effective strain have a greater probability of new bone formation, while regions of low effective strain lead to bone being resorbed in all femur defect regions.

We combined micro-CT imaging and micro-FE (Figures 1A,C) to determine mechanical stimulation in the microenvironment in 20 animals (10 loaded and 10 control) from a femoral defect loading study (Wehrle et al., 2019b). The control group was sham-loaded (0 N) for 5 min thrice weekly, and the loaded group was loaded according to the real-time finite element (rtFE) protocol indicated by Paul et al. (2021), resulting in the loads indicated in the Supplementary Material. We assessed the changes in bone volume, rates of bone formation, and resorption (Figure 2) and the mechanical environment in four regions, the cortical region, medullary region, peripheral region, and then the combination of all three (termed “all”) (Figure 1B). Mechano-regulation was assessed using two methods. The first entailed a conditional probability approach, whereby the conditional probability of a surface voxel forming, resorbing, or remaining quiescent was calculated as a function of the percentage of maximum effective strain in the region. Second, the area under the curve (AUC) of the receiver operating characteristic (ROC) was used to indicate the level of mechano-regulation, where the correct classification rate of each voxel at the following time point (a formation or a resorption voxel) for a given effective strain was determined.

### Longitudinal Bone Changes During Fracture Healing

**FIGURE 3:**
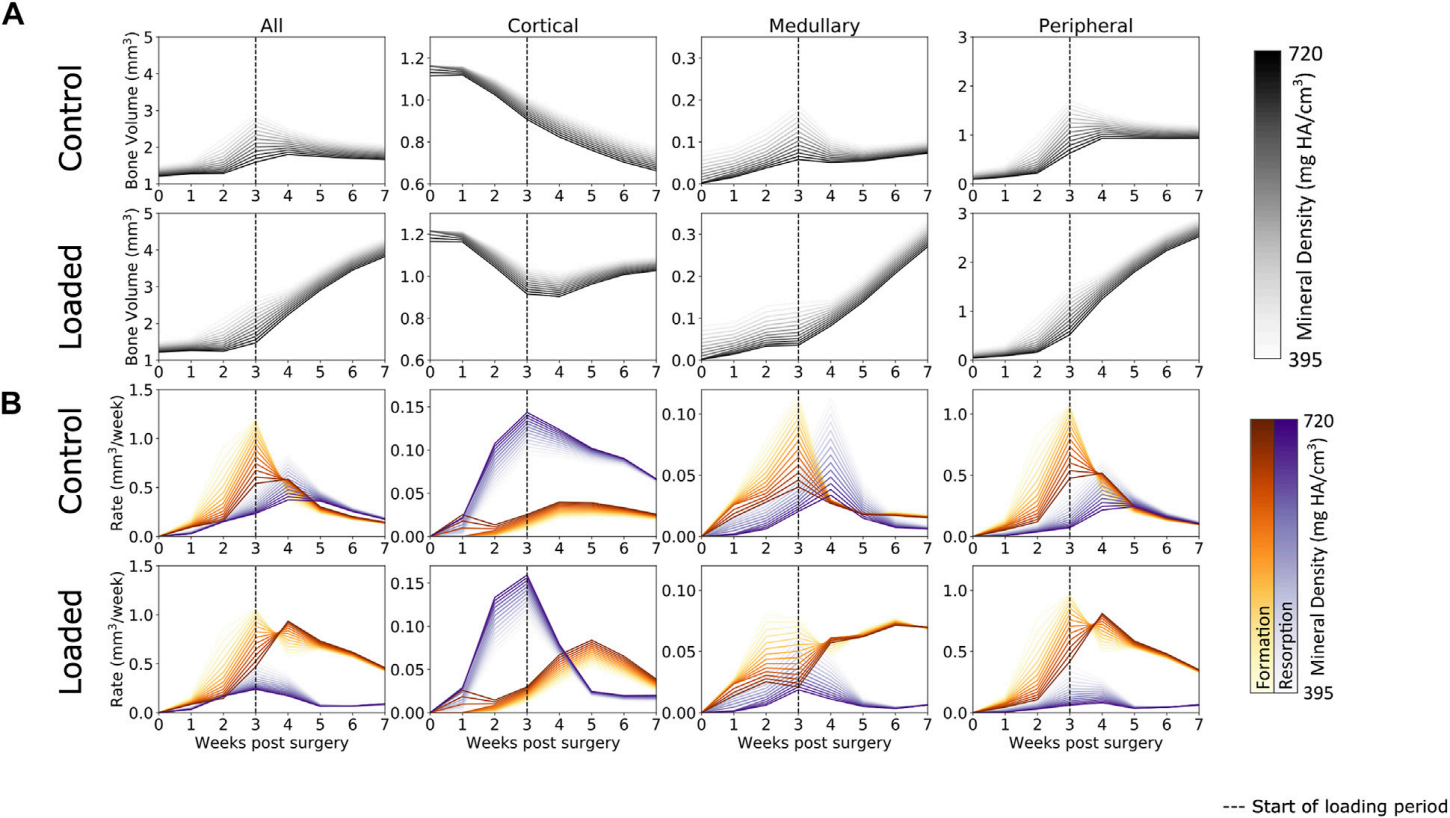
**(A)** Bone volume over time in femur defect regions. In both the loaded and control groups, lowly mineralized tissue starts to form at week 1. After week 3, mineralization occurs and the control group remodels away excess tissue, while the loaded group continues to form both lowly mineralized and mineralized tissue. A similar pattern is seen in the medullary and peripheral regions, while the cortical region sees substantial bone resorption, a process which is arrested by loading. **(B)** Formation and resorption rates over time. In all regions, peak formation occurs at week 3, while loading increases the amount of mineralized tissue forming at week 4. Similar patterns are seen in the medullary and peripheral regions, while resorption dominates the cortical region for the control group. Loading increases the rate of formation and decreases the rate of resorption in the loaded group’s cortical region.

Defect healing was observed to follow a typical pattern (Figure 3A) of bone formation, consolidation, and remodeling. Lowly mineralized bone tissue begins to form at week 1 and accelerates until week 3. By week 3, sufficient amounts of bone tissue will be formed for both the loaded and control groups to bridge the osteotomy gap for all mice but one in the control group (which bridged at week 4). Prior to week 3, all groups were treated the same, and from week 3 onward, the loaded protocol was implemented. At week 3, the control group had begun to display consolidation, whereby lowly mineralized bone tissue was mineralized, and the excess callus was remodeled away, approaching an equilibrium by week 7. Contrastingly, from the onset of loading in week 3, far greater bone formation was seen in the loaded group. By week 7, twice as much bone tissue was present in the loaded group compared to the control group. Lowly mineralized tissue was continuously forming, albeit at a decaying rate, but consolidation occurred, leading to far more bone tissue of all levels of mineralization in comparison to that in the control group. These patterns were mimicked in the bone formation and resorption rates (Figure 3B), where high rates of lowly mineralized tissue formation (up to ∼1 mm^2^ per week at week 3) preceded rates of highly mineralized tissue formation (up to ∼0.75 mm^2^ at week 4). In contrast with the control group, the loaded group expressed a higher peak formation rate of mineralized tissue (loaded group: ∼0.8 mm^2^ vs. control group: ∼0.5 mm^2^) at week 4 and suppressed resorption rates for all mineralization levels. In addition, the control group’s peak resorption rate occurred one week later than that of the loaded group.

When separated into three regions, several interesting patterns emerged. It was observed that behavior in both the medullary and peripheral regions was similar to the “all” region behavior. The original cortical region underwent significant resorption during the first 3 weeks of the healing period in both the loaded and control groups. Upon the application of load, bone resorption was arrested in the loaded group, despite the original cortical wall not being restored upon completion of the study.

### The Mechanical Environment During Fracture Healing

**FIGURE 4:**
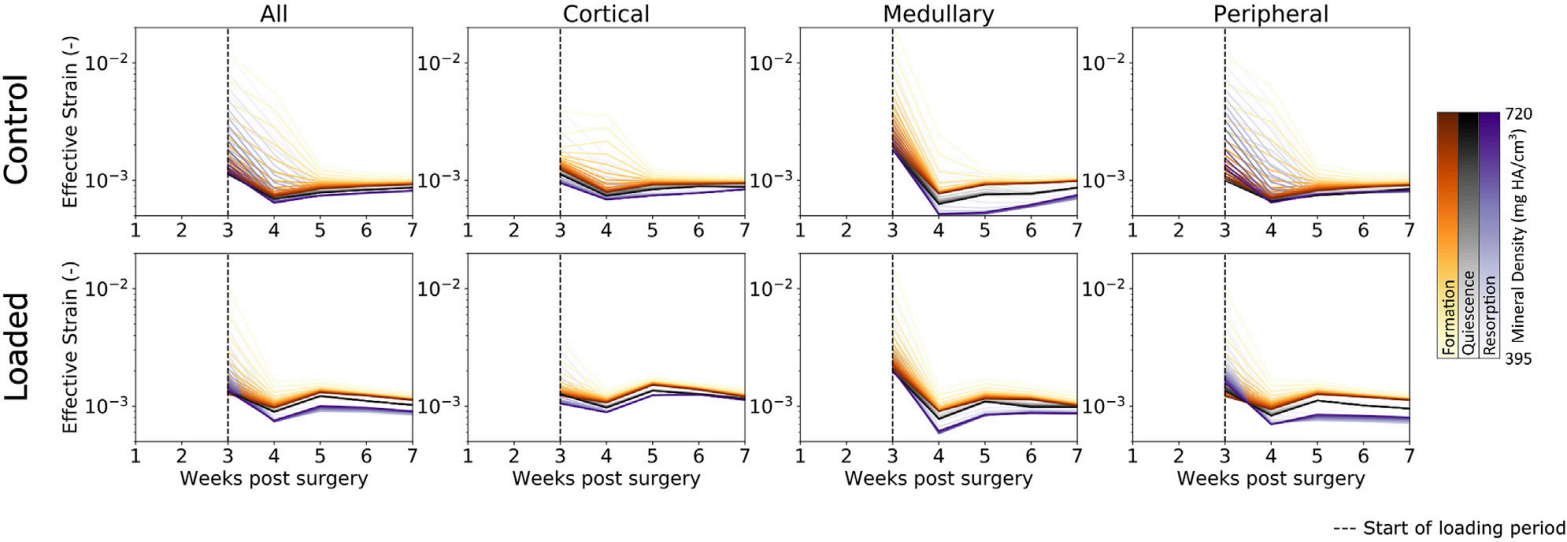
Longitudinal mechanical environment in femur defect regions. As lowly mineralized tissue further mineralized, the mechanical environment becomes more homogenous between bone of differing densities in both the control and loaded groups. For all densities of formation, the average effective strain was higher than for quiescent or resorption voxels, while resorption voxels displayed the lowest effective strain on average of the three possible changes.

As bridging occurs, effective strain consolidates within the range of mineralized tissues (Figure 4) (i.e., from low to high bone density), which was seen by reduction in the broad initial range of effective strain across multiple densities. This is due to the formation of a complete callus and the mineralization of lowly mineralized tissue. As more tissue forms and mineralizes, the organ-scale load is more evenly distributed throughout the tissues, such that extreme deformations of the lowly mineralized tissue can be avoided. Effective strains in the control group increased slightly throughout the observation period. However, for all time points, effective strain found in voxels of formation were higher than those in quiescent voxels, which in turn were higher than effective strain in resorption voxels. For the loaded group, the rtFE method led to effective strain increasing until all mice had reached the maximum load. Hence, effective strain peaked at week 5 and decreased to week 7, as the bone volume approached equilibrium, while the applied load remained constant. Dispersion of formation, quiescence, and resorption effective strain values were also observed, with greater degrees of dispersion particularly in the peripheral region.

In all regions, formation voxels of lower levels of mineralization were more likely to have higher effective strain than those of greater levels of mineralization. For the peripheral region, resorbed voxels of lower mineralization displayed lower levels of effective strain than those of greater mineralization. Quiescent voxels displayed no mineralization dependency of effective strain.

### Mechano-Regulation During the Post-Bridging Phase

Formation and resorption displayed clear mechano-regulatory behavior in both the loaded and control groups. The conditional probability of bone formation and resorption displays a logarithmic-like behavior. High effective strain increased the conditional probability of formation occurring rapidly at first and then gradually as effective strain further increased. For resorption, the conditional probability decreased quickly at first and then gradually as effective strain further increased.

**FIGURE 5:**
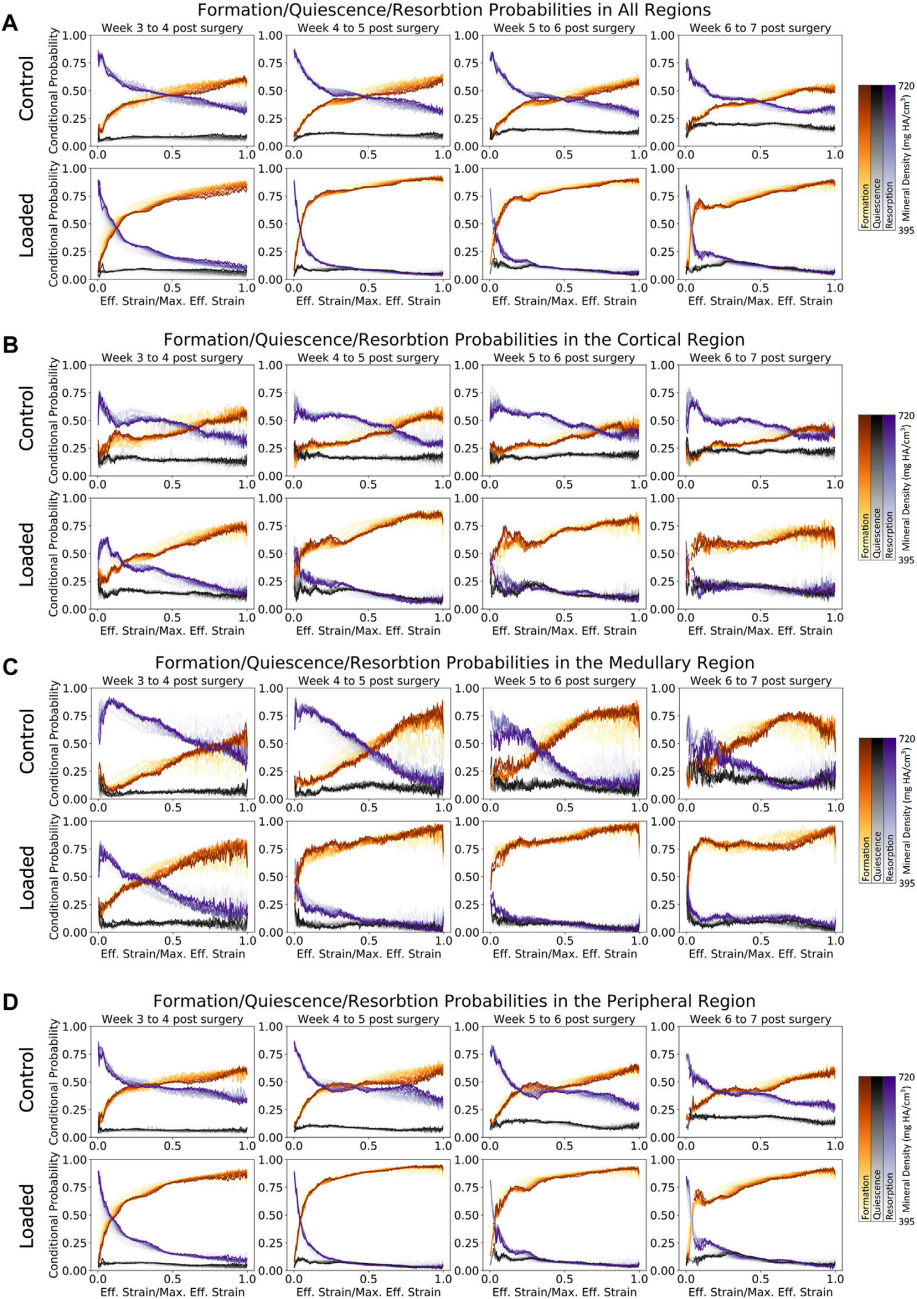
Conditional probability of formation/quiescence/resorption based on effective strain in femur defect regions. **(A)** Formation and resorption show clear mechano-regulation for all time points. **(B)** Cortical regions show less mechanosensitivity in early time points in the loaded group, while the control group shows that very high strains are required before formation is most likely to occur. **(C)** Similar to the cortical region, the loaded group shows increasing mechanosensitivity toward week 7. **(D)** In the peripheral region of the loaded group, it is evident that during the earlier time points, lowly mineralized tissue is more likely to form than highly mineralized tissue for voxels under effective strains up to half of the maximum effective strain.

As seen in Figure 5A, both the loaded and (to a lesser extent) control groups showed a clear relationship between the effective strain level and tissue mineral density throughout the post-bridging period. For formation voxels at week 3, voxels of lower mineralization were more likely to be formed for all effective strain values, while by week 7, higher effective strain values were more likely to lead to further mineralization of voxels. Mid-strain values appeared to lead to lower probabilities of resorption in lowly mineralized tissue than in tissue of greater mineralization, while for very high effective strain, this pattern was reversed. This general pattern was seen in all regions. However, in the cortical regions (Figure 5B), where the original cortex was remodeled away, the mechanosensitivity of the control group decreased substantially over time. Initially, the formation probability at maximum effective strain was 60% and decreased to 40% by week 7. A similar, but less drastic, decrease was seen in the loaded group from week 4 to week 7, where the probability for formation to occur decreased from 80% to roughly 65%. The medullary region (Figure 5C) displayed an increase in mechanosensitivity from week 3 until week 6 for both the control and loaded groups. This large degree of mechanosensitivity decreased from week 6 to week 7 in the control group, particularly for very high effective strain. In contrast, the loaded group maintained a high degree of mechanosensitivity until week 7. An additional change in mechanosensitivity was seen in the peripheral region (Figure 5D) for the final time point (week 6 to week 7) of the loaded group. For voxels of mid-to-lower effective strain, a lower conditional probability was observed in comparison with earlier time points.

**FIGURE 6:**
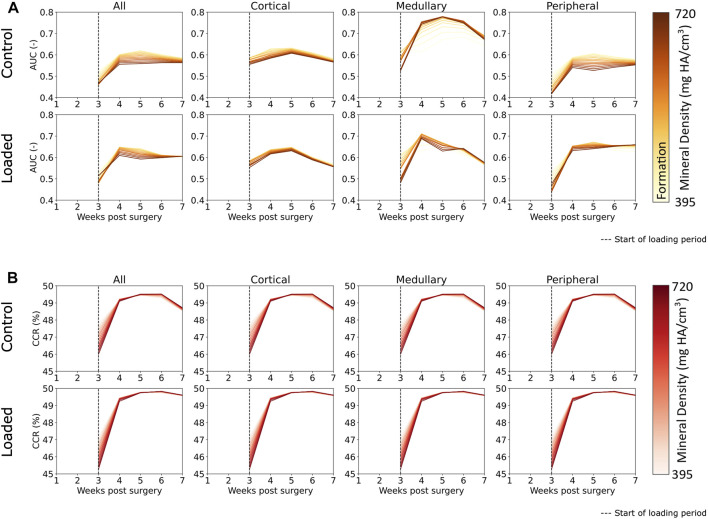
**(A)** Area under the curve (AUC) for classification of regions of formation and mineralization based on effective strain values in femur defect regions. All regions displayed effective strain as better than random predictor of formation. Effective strain acts as a better predictor for the mineralization of lowly mineralized tissue for all regions except the medullary region in the control group. **(B)** Correct classification rate of formation, resorption, and quiescent voxels show that extra-physiological loading increases predictability based on effective strain. The decrease of predictability from week 6 to week 7 was observed in the control group but not in the loaded group, and this indicates that the control group is closer to balanced remodeling by the end of the study at week 7 than the loaded group.

The AUC results indicate mechano-responsive behavior from week 3 onward (Figure 6), with an AUC value greater than 0.5 for formation over all densities in both the control and loaded groups. For the loaded group, in the medullary and peripheral regions, the AUC value from week 3 onward is higher for all resorption voxels (regardless of density) in comparison to formation voxels. Similar results are seen in the correct classification rate (CCR) for formation, quiescent, and resorption voxels (Figure 6). For all post-bridging time points, the CCR is greater than 33%. The lowly mineralized tissue displays a higher CCR in both the loaded and control groups until week 4, with lower starting values for the loaded group. From week 4 onward, the CCR for highly mineralized tissue was higher (∼49.5% in the control group and 49.75% in the loaded group). Peak CCR occurred at week 6 and then decreased in week 7. The decrease for the control group was larger than that in the loaded group. The CCR displayed similar patterns and values for every region.

The conditional probability of quiescence (Figures 5A–D) displayed independence from effective strain both locally (i.e., for all regions) and globally, hence demonstrating that quiescence is not mechano-regulated. This observation holds true for all mineralization levels of the tissue.

## Discussion

The purpose of this study was to investigate the relationship between effective strain in the microenvironment and the formation and resorption behavior of a loaded femoral defect model in mice. We combined longitudinal micro-CT scanning and micro-FE simulation to determine mechano-regulatory relationships between tissue-level effective strain and changes within bone tissue. High effective strain strongly increased the likelihood of bone formation, while low effective strain increased the likelihood of bone resorption. Our results align with results seen in bone adaptation models, providing support to the idea that the late reparative phase and remodeling phase exhibit similar behavior to conventional bone remodeling (Isaksson et al., 2009).

Similar to the study by Schulte et al. (2013); Malhotra et al. (2021), in a loaded vertebrae model of bone adaptation in mice, we observed an exponential relationship for formation and resorption; however, while this was generally observed in all regions, the cortical and medullary regions did not display such an evident relationship, appearing somewhat linear in the earlier time points. It is worth noting that the steepness of the initial exponential response increased as the study progressed. This indicates that the exponential response of formation and resorption to mechanical stimuli is a dominant function of remodeling behavior and not reparative behavior. Given that this exponential response develops earlier in the loaded group than in the control group, mechanical loading may be increasing the rate of transition from the reparative phase to the remodeling phase. This is also reflected in Figure 3B, where the resorption peaks of the loaded group occur earlier than those in the control group. Furthermore, the rate of mineralization and the formation peaks is higher in the loaded group than in the control group at week 4.

The novelties of our approach allow us to show that the mineralization dynamics are tightly interwoven with the formation and resorption of new packets of bone. Lowly mineralized bone displays a greater conditional probability to precede highly mineralized bone, particularly in the reparative phase. Assessing the AUC results (Figure 6), Scheuren et al. (2020) and Tourolle né Betts et al. (2020) showed similar AUC values for formation. However, in comparison with the study by Tourolle né Betts et al. (2020), our range of AUC values was more consistent in our data, which is most likely a result of better control of the mechanical environment due to extra-physiological loading. Mechanical loading appeared to increase the predictability of highly mineralized tissue formation as AUC values for highly mineralized tissue were comparatively higher in the loaded group for every region than in the control group. Formation in the medullary region in the control group displayed the highest level of mechanical control. The peak AUC was ∼0.75 at week 5 in the control group in comparison to peak AUC in the loaded group (week 4, ∼0.7). Formation of highly mineralized tissue was also better predicted from week 4 onward in the medullary region than in any other region. Extra-physiological loading did not increase the AUC values in the cortical region or all regions, indicating that the mere addition of extra load does not necessarily lead to increased mechanosensitivity, perhaps tying in to the concept of Frost et al.’s mechanostat (Frost, 1987), where, once a specific effective strain set point has been exceeded, additional mechanical stimulation does not further increase mechano-regulation. The CCR results, which additionally incorporate resorption and quiescence, show that mechanical loading does slightly increase the predictability of remodeling in every region. In particular, extra-physiological loading keeps predictability higher at week 7, indicating that the control group is closer to balanced remodeling (Tourolle né Betts et al., 2020) than the loaded group. The formation, resorption, and quiescence of lowly mineralized tissue are more predictable than those of highly mineralized tissue at week 3. The predictability of tissue changes increased in general from week 3 to week 4, with highly mineralized tissue increasing to a greater degree of predictability than lowly mineralized tissue. This observation is intuitive as more lowly mineralized tissue is present at week 3 than in the following weeks. The lower CCR at week 3 indicates that bone regeneration is less predictable, based on effective strain, than remodeling behavior.

The mapping of the response between local effective strain and the conditional probability for formation, quiescence, and resorption has great potential to aid *in silico* simulations. Many authors have built models for the prediction of defect healing (Isaksson et al., 2006; Geris et al., 2009; Isaksson et al., 2009). However, these models most often use theoretical or mathematical descriptions for the likelihood of voxels being remodeled under a particular load. These approaches, while built on global experimental observations and theories, do not use a locally derived relationship such as observed in our study. Therefore, our results can be used as a “mechano-stat” curve to provide experimentally supported probability to improve real-world legitimacy of healing simulations. The multidensity analysis approach aims to provide a continuum of relationships by categorizing the bone mineral density and related mechanical stimulation into far smaller discretization than previous approaches. While describing these overarching relationships is currently qualitative in its description, further efforts should start with a thorough analysis of these data in the context defect healing models and then move onto using such modeling and analysis techniques to work toward group and temporal quantitative interpretation of the AUC, ROC, and CCR results.

This work contains several limitations. Partial volume effects affect voxels on the boundary of bone and soft tissue, leading to artificially low grayscale values, hence affecting formation/resorption values for the lowest level of mineralization within the multidensity analysis. However, this limitation is largely addressed by the multidensity method, where shifts in grayscale values are captured within the binning approach. Another limitation is the description of the boundary conditions. Here, we made use of a simple superposition of a uniaxial load and a bending load derived from geometries and loading parameters of the external fixator, while the fixator–bone arrangement is under a dynamic load and hence, the mechanical response is dynamic, the range of mechanical stimuli reflecting that within the literature (Duda et al., 1998; Meakin et al., 2014; Razi et al., 2015; Liu et al., 2018; Wehrle et al., 2021). We, therefore, consider this static analysis sufficient for this study. As the bone´s stiffness increases with the progression of healing, the PEEK fixator’s stiffness becomes relatively smaller, leading to an increase in pin rotation. This compliance of the external fixator could lead to a larger bending load than the one we have used here. Capturing such large deformation would require extensive modeling and validation but could decrease error in this dataset. In addition, we have not performed a traditional intergroup statistical analysis as this study aims to move toward the analysis of mechano-regulatory relationships as a continuum and not limited by simple threshold-based analyses (within the limitation of the imaging modality). Such conventional loading experiment outcomes, highlighting bone volume changes and the impact of loading, are well described by Wehrle et al. (2019a); Wehrle et al. (2021). A final limitation that is worth noting is the lack of inclusion of the effect of the microstructure on mechanical stimuli. For example, our finite element models do not model strain amplification or any effects of the lacunae. Inclusion of such modeling would greatly expand the impact of this study but was beyond its scope.

In summary, we investigated the mechano-regulation of the post-bridging stages in a mouse femur defect loading model. Results show that increase in effective strain in the microenvironment lead to increased probability of formation and decreased probability of resorption. The inverse is also true; low effective strain increases the probability of resorption, while simultaneously decreases the probability of formation. In addition to this, quiescence is not mechano-regulated, displaying independence from the level of effective strain. We were able to confirm our hypothesis that high effective strain would lead to bone formation, while lower effective strain would lead to resorption. In addition, we were able to demonstrate that the mineralization process of the lowly mineralized bone is mechano-regulated and that this relationship is temporally dependent. This means that the mechanosensitivity of different densities of bone changes over time and, at certain time points, lower levels of mineralized bone are more likely to form, while high levels of mineralized bone are more likely to be resorbed. This work sets the stage for three future investigations. First, extension of mechanical loading protocols and mechano-regulatory analysis into the pre-bridging phase will elucidate the early stages of fracture healing, potentially giving rise to the possibility of improved interventions. Second, the established conditional probability relationship can act as an input into *in silico* models, allowing accurate mechano-regulatory relationships within bone healing and remodeling simulations. Finally, the translation of this mechano-regulatory behavior down to a cell scale, *via* the incorporation of either high-resolution scanning or histological approaches, would improve our ability to link the organ scale loading to cell-scale responses, allowing further understanding of the osteocyte–osteoblast–osteoclast mechanobiological relationship.

## Materials and Methods

### *In vivo* Experiments and Loading Protocol

Details regarding animal experiments and mechanical-loading protocol are mentioned by Wehrle et al. (2021) and Paul et al. (2021). All animal procedures were approved by the relevant authorities (license number: 36/2014, Kantonales Veterinäramt Zürich, Zurich, Switzerland). All methods were carried out in accordance with the ARRIVE guidelines and the Swiss Animal Welfare Act and Ordinance. All mice (20 female, and C57BL/6J) were acquired from Janvier (Saint Berthevin Cedex, France) at an age of 12 weeks and were housed in the ETH Phonemics Center animal facility under a 12:12 h light–dark cycle, maintenance feed (3,437, KLIBA NAFAG, Kaiseraugst, Switzerland), five animals/cage for 8 weeks until surgery. Female animals were used to ensure consistency with previous studies. All animals at 20 weeks of age underwent osteotomies on the right femur with a 0.66-mm Gigli wire by the same veterinarian. The details can be found in the study by Wehrle et al. (2019a). The mice were divided into two groups, the control/sham loading group (*n* = 10) and the loaded group (*n* = 10). Post surgery, they were housed with 2–3 animals per cage. For all surgeries and micro-CT scans, the animals were anaesthetized with 5% isoflurane/oxygen for inductance and maintained at 1–2% isoflurane/handling.

Mechanical loading was performed thrice weekly (10 Hz loading frequency, 300 s loading time, and 3,000 cycles) from week 3 onward. RtFE (Paul et al., 2021) was used to determine the loading parameters, which are contained in Supplementary Table S1 in the Supplementary Material.

### Imaging, Pre-Processing, Masking, and Multidensity Finite Element Analysis

Imaging was performed on a (Scanco Medical, Brüttisellen, and Switzerland) reconstructed micro-CT image at a nominal resolution of 10.5 um. The scanned region required two stacks of 211 voxels each and had an imaging time of 15 min. All animals were scanned weekly from week 0 (post operation) until week 7 (post operation). All images for each time point of each mouse were registered to the baseline image (week zero) of that particular mouse. Pre-processing entailed the extraction of the relevant sub volume (reducing the image size to 300 × 300 × 180 voxels), Gaussian filtration (σ = 1.2, support = 1), and binning gray values using a multidensity approach proposed by Tourolle né Betts et al. (2020).

Masks (Figure 1B) were generated with a ray tracer approach as performed by Tourolle né Betts et al. (2020) from each baseline image. The original cortices were extracted by thresholding all tissues above a bone mineral density of 645 mg HA/cm^3 18^, while the medullary region (marrow cavity) and the peripheral region (everything else) were extracted from the remaining regions.

For mechanical simulations, the binned grayscale values were converted from density (mg HA/cm^3^) to Young’s moduli (GPa) on a per voxel basis. The regions of soft tissue were set to a Young’s modulus of 0.003 GPa (Claes and Heigele, 1999) and the marrow cavity of the femur was capped with a plate of 20 GPa, preventing edge effects due to the soft tissue found lying on the top slice of the finite element mesh. A linear micro finite element (micro-FE) solver, parasol (Flaig and Arbenz, 2011), was then used to solve the finite element mesh. For the uniaxial loading case, 1% compressive displacement was applied to the top slice in the axial direction, and the bottom-most slice was fixed (only in the axial direction). For the bending case, the center of bending was determined *via* a center of mass calculation found in the SciPy library (Virtanen et al., 2020), and the bending load was centered on the axis of loading from the loading machine and deformed by 1% at the furthest edges of the mesh. The Swiss National Supercomputing Center (CSCS) was used to solve each finite element simulation, requiring roughly 2 min per image.

### Estimation of Mechanical Stimulation

The local *in vivo* mechanical environment was described using effective strain, calculated as described by Pistoia et al. (2002). The results of the simulations were appropriately scaled based on the assumed loading parameters using the following ratios:

$$\varepsilon_{axial\_actual}=\;\frac{F_{axial\_applied}}{F_{axial\_resultant}}\varepsilon_{axial\_simulation}.$$

Or for a bending moment:

$$\varepsilon_{bending\_actual}=\;\frac{F_{bending\_applied}}{F_{bending\_resultant}}\varepsilon_{bending\_simulation},$$

where $\varepsilon_{simulation}$ is the effective strain result of the simulation (based on the 1% displacement), is the sum of reaction forces of all the nodes of the upper most surface. *F_resultant_*, the reaction forces occur as a result of the displacement applied to the nodes. $F_{applied}$ is the selected force (i.e., a force provided by a mechanical stimulation machine) and $\varepsilon_{actual}$ is the strain under the applied force. Bending moments were determined using length of the implant pins as the moment (10 mm) and the loading force from the machine (described in Supplementary Material, ranging from 8 to 16 N). Axial forces were taken as the loading force, and the bending and axial loads were superimposed upon one another. All control mice were assumed to have a uniaxial load of 10 N as described previously (Tourolle né Betts et al., 2020).

### Analysis of Bone Volume and Formation, Quiescence, and Resorption Rates

Bone volume was calculated by counting the number of voxels above an increasing density threshold. The threshold densities ran from 395 mg HA/cm^3^ to 720 mg HA/cm^3^ in incremental steps of 25 mg HA/cm^3^. Regions of formation, quiescence, and resorption were calculated by the binary difference between an image at a given time point and neighboring image at a preceding time point to establish their respective rates of change. Voxels present in both images were classified quiescent, present in the most current time point were classified as formation, and only present in the preceding time point were classified as resorption. Masks were then formed to describe these regions.

### Statistical Analysis of Local Mechano-Regulation

The scaled results of the micro-FE simulation in terms of effective strain were used as a measure of mechanical stimuli. The mechanical stimuli as calculated before were mapped to each voxel. The mean strain at each time point was calculated on a per group basis. Mean effective strain for each density band was calculated. The conditional probabilities for formation, quiescence, and resorption were calculated to occur for a given value of effective strain (as per Schulte et al. (2013)) at a given bone tissue density. The surface effective strain values were normalized to the 99^th^ percentile effective strain in the whole simulation region to ensure that simulation artifacts did not affect the analysis.

The area under the curve (AUC) of a receiver operating characteristic ROC curve was used to assess the performance of the particular effective strain value as a predictor of formation and mineralization. An AUC greater than 0.5 implies that the change in voxel (either a new voxel forming, or an existing voxel mineralizing) is associated with mechanical stimulation. An AUC of 0.5 indicates no relationship between the mechanical stimuli and the voxel, while below 0.5 indicates an inverse relationship between mechanical stimuli and voxel behavior.

As prediction of formation, quiescence, and resorption from preceding strain is a multiclass classification problem, AUC/ROC approaches could not be used. Hence, the mechanobiology of callus remodeling classification approach developed by Tourolle né Betts et al. (2020) was applied. This approach uses two thresholds, one upper threshold to classify the sites of formation (i.e., bone density values above were considered to have formed) and one lower to classify sites of resorption (i.e., bone density values below were considered to be resorbed). Any value between these thresholds was classified as quiescence. The ground truth was determined by comparing sequential time-lapsed images for formation, quiescent, and resorption regions. Similar to the ROC, thresholds were swept through the range of possible effective strain values to derive a matrix corresponding to whether a voxel was classified correctly based on the two particular thresholds. This correct classification rate (CCR) was determined for each mouse, at each time point post bridging (week 3 onward) and at each bone density level (i.e., from 395 mg HA/cm^3^ to 720 mg HA/cm^3^ in steps of 25 mg HA/cm^3^). As three states are possible (formation, quiescence, and resorption), the maximum CCR would need to be greater than 33% to indicate mechano-regulation within the tissue.

## Data Availability

The raw data supporting the conclusion of this article will be made available by the authors, without undue reservation.

## Supplementary Material

Supplementary Table S1 Loading parameters for the loaded group.
