# Supplementary material for: Tissue-Level Regeneration and Remodeling Dynamics are Driven by Mechanical Stimuli in the Microenvironment in a Post-Bridging Loaded Femur Defect Healing Model in Mice
Source: Front Cell Dev Biol. 2022 May 24;10:856204. doi: 10.3389/fcell.2022.856204 (PMC9171432; doi:10.3389/fcell.2022.856204)
Supplement: Supplementary file 1 [file Table1.docx]

Table 1: Loading Parameters for the loaded group

| Mouse | Week 3 | Week 4 | Week 5 | Week 6 | Week 7 |
| --- | --- | --- | --- | --- | --- |
| 1 | 8 N | 14 N | 16 N | 16 N | 16 N |
| 2 | 8 N | 12 N | 16 N | 16 N | 16 N |
| 3 | 12 N | 16 N | 16 N | 16 N | 16 N |
| 4 | 8 N | 14 N | 16 N | 16 N | 16 N |
| 5 | 12 N | 16 N | 16 N | 16 N | 16 N |
| 6 | 10 N | 12 N | 16 N | 16 N | 16 N |
| 7 | 8 N | 16 N | 16 N | 16 N | 16 N |
| 8 | 10 N | 16 N | 16 N | 16 N | 16 N |
| 9 | 10 N | 16 N | 16 N | 16 N | 16 N |
| 10 | 12 N | 16 N | 16 N | 16 N | 16 N |
